# Decorative Magnolia Plants: A Comparison of the Content of Their Biologically Active Components Showing Antimicrobial Effects

**DOI:** 10.3390/plants9070879

**Published:** 2020-07-11

**Authors:** Petra Lovecká, Alžběta Svobodová, Anna Macůrková, Blanka Vrchotová, Kateřina Demnerová, Zdeněk Wimmer

**Affiliations:** 1 Department of Biochemistry and Microbiology, University of Chemistry and Technology in Prague, Technická 3, 166 28 Prague 6, Czech Republic; svobodoz@vscht.cz (A.S.); anna.macurkova@vscht.cz (A.M.); blanka.vrchotova@vscht.cz (B.V.); demnerok@vscht.cz (K.D.); 2 Department of Chemistry of Natural Compounds, University of Chemistry and Technology in Prague, Technická 5, 166 28 Prague 6, Czech Republic; wimmerz@vscht.cz; 3 Isotope Laboratory, Institute of Experimental Botany, Czech Academy of Sciences, Vídeňská 1083, 142 20 Prague 4, Czech Republic

**Keywords:** Magnolia genus, Staphylococcus aureus, medium-polar extract, neolignan, magnolol, obovatol, honokiol, antimicrobial activity

## Abstract

*Magnolia* plants are used both as food supplements and as cosmetic and medicinal products. The objectives of this work consisted of preparing extracts from leaves and flowers of eight *Magnolia* plants, and of determining concentrations of magnolol (1 to 100 mg·g^−1^), honokiol (0.11 to 250 mg·g^−1^), and obovatol (0.09 to 650 mg·g^−1^), typical neolignans for the genus *Magnolia*, in extracts made by using a methanol/water (80/20) mixture. The tested *Magnolia* plants, over sixty years old, were obtained from Průhonický Park (Prague area, Czech Republic): *M. tripetala* MTR 1531, *M. obovata* MOB 1511, and six hybrid plants *Magnolia × pruhoniciana*, results of a crossbreeding of *M. tripetala* MTR 1531 with *M. obovata* MOB 1511. The identification of neolignans was performed by HRMS after a reversed-phase high-performance liquid chromatography (RP-HPLC) fractionation of an extract from *M. tripetala* MTR 1531. The highest concentrations of neolignans were found in the flowers, most often in their reproductive parts, and obovatol was the most abundant in every tested plant. The highest concentrations of neolignans were detected in parent plants, and lower concentrations in hybrid magnolias. Flower extracts from the parent plants *M. tripetala* MTR 1531 and *M. obovata* MOB 1511, flower extracts from the hybrid plants *Magnolia × pruhoniciana* MPR 0271, MPR 0151, and MPR 1531, and leaf extract from the hybrid plant *Magnolia × pruhoniciana* MPR 0271 inhibited growth of *Staphylococcus aureus*.

## 1. Introduction

*Magnolia* plants have been grown for decorative purposes in Europe and America, while in Asia these plants (especially *M. officinalis* and *M. obovata*) have been used in traditional medicine for centuries in order to treat gastrointestinal disorders, anxiety, cough, allergies, or asthma [1].

The biological and healing effects of plants appear due to their secondary metabolites, which protect plants from the effects of the external environment. Knowledge about the medicinal effects from traditional medicine has resulted in a great interest in determining the plant metabolites that display biological effects for their expected applications—not only for the preparation of medicinal products, but also for their use as food supplements or biopesticides [2,3,4,5].

Antimicrobial, antioxidant, antitumor, and neuroprotective effects or cardiovascular protective effects have been demonstrated with many secondary metabolites of the genus *Magnolia* [5,6,7]. Those authors summarized the results of 58 selected studies on the biological activity of *Magnolia* plants. The effects of the substances were the most evident in the area of metabolism (37.5%), in the central nervous system (25%), and in cardiovascular treatment (20.8%).

At least 225 biologically active substances, among which are lignans, neolignans, terpenoids, and alkaloids, have been described in the *Magnolia* genus [2,6,7]. The most commonly studied secondary metabolites of this genus include neolignans, namely magnolol, honokiol, and obovatol. Ninety percent of the biological effects of *Magnolia* plants are mediated by the most frequent plant secondary metabolites, i.e., magnolol and honokiol [1,8].

The increase in the concentration of secondary metabolites with the growing age of the plant occurs mainly in its bark, which plays a significant role in the production of medicinal products from *Magnolia*. To prepare such products from the bark of *M. officinalis*, the plant must be older than 15 years. However, subsequent peeling of the bark, which can also be obtained from the root of the plant, may result in a threat or even in an extermination of the plant source.

In recent years, and not only for the above-mentioned reason, investigation has been focused on analyzing quantities of secondary metabolites in parts of *Magnolia* plants other than its bark [6,7]. Leaves should be available sources of useful substances in these plants, which could be subject to danger if they are torn apart, in order to protect them from damage that could occur during the harvesting of their bark. However, magnolol and honokiol were found in only one-fifth quantities in comparison to their concentration in the bark [7,9,10,11].

The objectives of this work consisted of (a) preparing extracts from leaves and flowers of eight *Magnolia* plants, (b) analyzing the concentration of magnolol, honokiol, and obovatol, neolignans typical for the genus *Magnolia*, obtained by extraction using a methanol/water (80/20, *v/v*) mixture and subsequent extraction of the organic layer by chloroform, and (c) determining the antimicrobial effects of the extracts. The studied plant material consisted of *Magnolia* plants that were over sixty years old from Průhonice Park: the parent plants *M. tripetala* MTR 1531 and *M. obovata* MOB 1511 and six hybrids resulting from their crossbreeding, *Magnolia × pruhoniciana*.

## 2. Results

**Figure 1:**
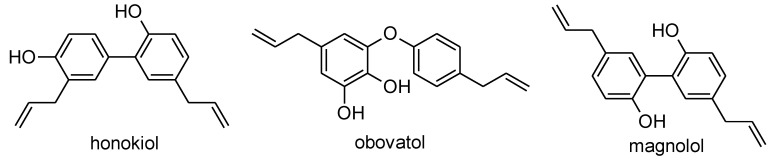
The structures of honokiol, obovatol, and magnolol.

The extract from *M. tripetala* MTR 1531 flowers, made by using a methanol/water (80/20, *v/v*) mixture and subsequent extraction of the organic layer by chloroform, was fractionated by reversed-phase high-performance liquid chromatography (RP-HPLC). Six elution zones were detected in the chromatograms, showing their retention times at 6.3 min, 44.1 min, 45.5 min, 46.0 min, 47.5 min, and 48.0 min. These fractions were subjected to a high-resolution mass spectrometry analysis (UPLC-QTOF HRMS/MS analysis). The investigated neolignans were identified in the only three fractions (fraction 2 (honokiol), fraction 3 (obovatol), and fraction 4 (magnolol); Figure 1). The remaining three fractions were not identified (Table 1).

The identification of magnolol and honokiol was performed by comparing the fragmentation of their mass spectra with the records in the METLIN online database. The mass spectrum of obovatol was not available from the METLIN database; however, this neolignan was identified based on data published in a paper [12], in which the fragmentation spectrum of obovatol was published.

On the basis of the calibration curve for the identified substances, the concentrations of the monitored neolignans in individual extracts were measured using an RP-HPLC method. A summarizing evaluation of the concentrations of honokiol, obovatol, and magnolol in individual parts of plants is given in Table 2.

In our investigation, we studied the content of neolignans in leaves and flowers of *Magnolia* plants, and we found that the flowers of the tested *Magnolia* plants were richer in neolignans than the leaves. The reason for the higher concentration of neolignans in *Magnolia* plant flowers may be based on their aromatic properties attracting pollinators. The aromatic and spicy aroma of *Magnolia* plant flowers is attributed to magnolol and honokiol [13].

A common feature of hybrid *Magnolia* plants and one of the parent plants, *M. obovata* MOB 1511, was the presence of obovatol, which was a major component in the flowers. Obovatol was identified as the main component of *M. obovata* leaves [14], and it was also confirmed by our measurements, resulting in a finding that *M. obovata* MOB 1511 leaves contained the largest amount of obovatol of the three neolignans studied.

The flowers of some *Magnolia* plants contained significantly higher quantities of neolignans compared to the reported concentrations of these substances in the bark of *Magnolia* plants. Scientific literature has reported that the *M. officinalis* bark extracted with ethanol (99.5%) contained 75.2 mg·g^−1^ of magnolol and 19.1 mg·g^−1^ of honokiol after HPLC analysis [5]. The *M. obovata* bark extracted with methanol contained 3.3 mg·g^−1^ of obovatol [1,15]. In our measurements, the flower (flower petals and sexual parts of the plants together) of *M. tripetala* MTR 1531 contained 183.07 mg·g^−1^ of magnolol and 483.08 mg·g^−1^ of honokiol, whereas the *M. obovata* MOB 1511 flower contained 921 ± 138.15 mg·g^−1^ obovatol. In another study on the content of secondary metabolites in *Magnolia* plants, the concentration of magnolol and honokiol in the leaves taken from seventeen plants of *M. officinalis* var. *biloba* grown in different regions of China was investigated. This plant is one of the subspecies, known to *M. officinalis*, and it is also used in traditional medicine. The mean content of the substances in the leaf samples was determined by HPLC analysis of methanol extracts as 0.23 mg g^−1^ of magnolol and 1.14 mg g^−1^ of honokiol [16].

The *Magnolia* leaves analyzed in our work showed higher concentrations of the studied compounds than it was found by other authors in the *Magnolia* leaves [16]. The highest concentration of the studied compounds measured in *M. tripetala* leaves was found as 53.61 mg·g^−1^ of magnolol and 191.62 mg·g^−1^ of honokiol.

The concentration of neolignans in hybrid plants of *Magnolia × pruhoniciana* has not yet been documented, and our work resulted in a new finding that parent plants contain considerably larger amounts of observed neolignans than hybrid plants. For example, the flowers of *M. tripetala* contained more than ten times the amount of honokiol compared to hybrid *Magnolia* flowers. Yet, when compared to the content of neolignans in *M. officinalis* bark [5], the *Magnolia × pruhoniciana* hybrids appear to be a rich source of honokiol and obovatol, and therefore a more detailed analysis of these plant parts is appropriate due to the possible content of other useful metabolites, not yet known or studied [17,18,19].

One of the properties of *Magnolia* plants is their antimicrobial effect, which was also one of the objectives studied in our work. The tested bacterium *Staphylococcus aureus* is one of the human pathogens causing life-threatening infectious diseases, such as pneumonia, or serious skin diseases with subsequent toxic shock syndrome. With the increasing resistance of bacteria (including *S. aureus*) to antibiotics, other possible ways of treatment have been intensively sought [9,20].

Antimicrobial activity values were measured for individual extracts, i.e., mixtures of the compounds contained therein. Substances in extracts can interact with each other, often acting in a synergy. The synergic effect of magnolol and honokiol has already been reported in the literature [1].

In most of the extracts tested, the concentration of 125 μg·mL^−1^, in some cases a concentration of 62.5 μg·mL^−1^, showed the highest inhibitory activity. The inhibition coefficients of these concentrations of individual extracts are given in Table 3. Leaves of some plants (e.g., leaves of *Magnolia × pruhoniciana* MPR 0271) also achieved high growth inhibition of the tested bacteria at a concentration of 125 μg·mL^−1^.

As shown in Table 3, only extracts from flowers and, in one case, from leaves inhibited the growth of *S. aureus* by 100%, and it was possible to determine minimum inhibitory concentration (MIC) and to calculate IC_50_ values. Among the hybrid *Magnolia* plants, 100% inhibition of bacterial growth was caused by extracts from the flowers of *Magnolia × pruhoniciana* MPR 0131, 0151, and 1531 plants and *Magnolia × pruhoniciana* MPR 0271 leaves, and, specifically, the extracts from the sexual parts of the flowers of *M. obovata* MOB 1511, and the two tested parts of the flowers of *M. tripetala* MTR 1531. MIC and IC_50_ values were further determined for the tested extracts. The results are summarized in Table 4.

A study by Kim [20] investigated the antibacterial effect of magnolol from *M. officinalis* bark extract against methicillin-resistant *S. aureus* (MRSA). Magnolol was obtained by an HPLC separation of the extract. The study described its promising effects and concluded with the potential use of magnolol in conjunction with antibiotics, which could lead to a development of new drugs against MRSA infection [20].

In our work, the antibacterial activity of extracts from individual plant parts of *Magnolia* was studied, but not that of individual neolignans. Thus, the effects observed cannot be attributed to a particular compound. Extracts from the sexual parts of parent plants MOB 1511 showed MIC values with respect to *S. aureus* of up to 40.1 μg·mL^−1^. The MIC values with respect to this bacterium, taken from the literature and in which the *M. officinalis* bark extract was tested, were reported to be about 20 μg·mL^−1^ [21].

## 3. Conclusions

Our study showed that the tested *Magnolia* blossom extracts contained higher concentrations of honokiol and obovatol than those reported in *M. officinalis* and *M. obovata* bark samples commonly used, from which a number of products are currently being prepared. These extracts very effectively inhibited the growth of *S. aureus*. A synergic effect of the studied plant products in the inhibition of growth of *S. aureus* was also observed. The beautiful flowers of decorative *Magnolia* plants containing suitable biologically active substances have proven their potential for practical pharmacological application and, therefore, the very useful reason to plant these flowers, in addition to their role of providing pleasure and a garden decoration in European areas.

## 4. Experimental Section

### 4.1. Plant Material

The plant material described above and used included parent plants (designated MTR 1531 and MOB 1511) and six hybrid *Magnolia* plants (designated MPR). The plants came from the Průhonice Park (49°59’25.81” N, 14°32’41.23” E) in the Czech Republic.

Hybrids were created by the common crossbreeding of *M. tripetala* and *M. obovata*, i.e., pollination of the flowers of one species with the pollen of another. Crossbreeding was done in the Průhonice Park by Viktor Keskevič before 1952.

### 4.2. Preparation of Extracts

Leaves, petals, and sexual parts of the flowers were taken from each *Magnolia* plant separately (10 flower pieces and the leaves of 3 trees). These plant parts were treated by freeze-drying (8.4 Pa, −54 °C) and then ground. A smaller amount was weighed out from each of the *Magnolia* portions treated (1.5 g for most plants) and diluted with 80% methanol. The homogenization of the material for subsequent extraction was performed by disintegration (130 MPa) of these suspensions using the ONE SHOT MODEL homogenizer (Constant Systems Ltd., Daventry, UK).

After disintegration, the suspensions from leaves, petals, and sexual parts of the flowers of each plant were washed with water six times in succession and the pellets obtained were resuspended in 80% methanol. The pellets and solutions were further processed according to a standardized procedure for extracting secondary metabolites with different organic solvents, pH, and polarity [22]. The extracts obtained were evaporated using a CentriVap refrigerated rotary concentrator (LABCONCO, Kansas City, MO, USA) (35 °C), dried, weighed, and diluted with 100% methanol to stock solutions for subsequent measurements.

### 4.3. RP-HPLC Analysis

Magnolol, honokiol, and obovatol (Figure 1) were identified, and quantified in the extracts made using a methanol/water (80/20, *v/v*) mixture, obtained after acidification (to pH = 2), and subsequent extraction of the organic layer by chloroform.

Identification of these three compounds was performed by high-resolution mass spectrometry. Identification of substances was performed on a 6560 Ion Mobility Q-TOF system (Agilent Technologies, Santa Clara, CA, USA). Aliquots (2 μL) of extracts were sprayed into an Agilent Infinity 1290 liquid chromatograph (Agilent Technologies, Santa Clara, CA, USA) with an Acquity UPLC BEH C18 (100 × 2.1 (i.d.) mm, particle size 1.7 μm) analytical column (Waters Corporation, Milford, MA, USA). The column temperature was 60 °C, and the flow rate was set up to 0.35 ml·min^−1^.

The mobile phase A consisted of 5 mM ammonium formate in a mixture of methanol/water (95/5, *v/v*) containing 0.1% of formic acid. The mobile phase B consisted of 5 mM ammonium formate in an isopropyl alcohol/methanol/water (65/30/5, *v/v*) mixture, containing 0.1% of formic acid.

The substances were detected based on the exact mass (*m/z*) of their [M-H]^–^ ion, the isotopic profile in the MS1 mass spectrum, and the specific fragmentation spectrum (the presence of specific fragments and their representation in the spectrum). A quadrupole/fly-through analyzer (Agilent Technologies, Santa Clara, CA, USA) was used for detection. Measured fragmentation spectra of substances, at collision energy (AutoMS/MS; 20 eV), were compared with the records in the METLIN online database and in the literature. Isolated pure magnolol, honokiol, and obovatol were used for calibration purposes.

Their concentration in individual extracts was analyzed by reversed-phase high-performance liquid chromatography (RP-HPLC). Samples (0.5 mg·mL^−1^; 10 μL) were injected into an Agilent 1100 liquid chromatograph with a Discovery^®^ C−18 (250 × 4.6 (i.d.) mm; particle size 5 µm) chromatographic separatory column (Aldrich, St. Louis, MO, USA). Methanol and a solution of 10 mM formic acid in water were used as mobile phase (Table 5).

Each individual measurement took 80 min with the mobile phase flow rate of 1 mL·min^−1^. The UV spectra were taken on a diode array detector (DAD) (Life Science UV/VIS Spectrophotometer DU 730, Beckman Coulter, Carlsbad, CA, USA) at 220 and 280 nm.

Fractions were prepared for identification of magnolol, honokiol, and obovatol by high-resolution mass spectrometry, and then the concentration of neolignans in the extracts from plant parts of the tested *Magnolia* plants was determined. For calibration purposes, isolated pure magnolol, honokiol, obovatol were used as reference compounds.

### 4.4. Antimicrobial Activity of Extracts

The spectrophotometric method was used for the determination of antimicrobial activity. The optical density (OD) of individual suspensions containing the tested extracts and the bacterium *Staphylococcus aureus* was measured at 625 nm.

The result of the method was the dependence of OD on the time–growth curve of the microorganism, which was influenced to some extent by added test extracts.

*S. aureus* was cultured on TSA (37 °C) overnight. Then, one colony was picked up and diluted with Ringer’s solution to OD_625_ = 0.1. The prepared suspension, containing approximately 2 × 10^9^ CFU·mL^−1^, was further diluted several times. Each well of the microtiter plate then contained 5 × 10^4^ CFU.

From the stock solutions of medium-polar extracts at a concentration of 10 mg·mL^−1^, aliquot parts of 250 µg·mL^−1^ solutions were prepared by dilution with Mueller–Hinton broth 2 medium, which was prepared with a concentration of 500 µg·mL^−1^. Solutions of medium-polar extracts from leaves, petals, and sexual parts of flowers were pipetted to the microtiter plate, at concentrations of 0 to 125 μg·mL^−1^. The prepared suspension of *S. aureus* (50 µL) was pipetted into each well.

The OD was measured every hour in the period of 24 h at a growth temperature of 35 °C, i.e., at the temperature recommended by the CLSI for determining the antimicrobial activity of the substances. Growth inhibition of microorganisms was evaluated using Equation (1):

*Inhibition = (*1 − (∆A_fraction_/∆A_control_))*100 (1)

*∆A_fraction_*—the OD difference at the beginning and at the 18th hour of the measurement of the antimicrobial samples,
*∆A_control_*—the OD difference at the beginning and at the 18th hour of the measurement for the unaffected microorganism.

A solution of the antibiotic vancomycin at a concentration of 0.1 mg·mL^−1^ was used as a positive control.

The measurement was performed in four parallels, and the standard deviation was calculated. Data were analyzed using the statistical functions of SigmaStat 3.0 (SPSS Inc., Chicago, IL, USA). If in a one-way analysis of variance test a significant F-value of *p* < 0.05 was obtained, a Dunnett’s multiple comparison test between the treated and control groups was conducted. The significance level was established at *p* < 0.05 for all statistical tests. An IC_50_ calculator from AAT Bioquest^®^ was used to calculate the IC_50_ values (https://www.aatbio.com/tools/ic50-calculator/).

## Figures and Tables

**Table 1:** Identification and retention times of compounds in the fractions 1–6.

Fraction	RT ^a^	Identified Substance	Main Mass Fragments (*m/z*)
1	6.3 min	unidentified	-
2	44.1 min	honokiol	209.0597; 224.0844; 250.0999
3	45.5 min	obovatol	133.0657, 164.0478. 240.0775
4	46.0 min	magnolol	223.0785; 245.0968, 247.1124
5	47.5 min	unidentified	-
6	48.0 min	unidentified	-

^a^ RT = approximate retention time of collecting the fractions by RP-HPLC.

**Table 2:** Concentrations of honokiol, obovatol, and magnolol in the given parts of the individual plants ^a^.

	Plant	Part of Plant	Honokiol (mg·g^−1^ Dry Mass)	Obovatol (mg·g^−1^ Dry Mass)	Magnolol (mg·g^−1^ Dry Mass)
*Magnolia hybrid plants*	MPR 0131	L	3.64 ± 0.04	0.68 ± 0.05	1.01 ± 0.08
		FP	7.25 ± 0.01	8.98 ± 0.2	2.95 ± 0.00
		SP	47.43 ± 7.11	92.13 ± 13.82	25.81 ± 3.87
	MPR 0151	L	2.01 ± 0.02	0.90 ± 0.01	0.76 ± 0.01
		FP	13.01 ± 1.95	24.59 ± 3.69	6.00 ± 0.90
		SP	35.95 ± 5.39	80.67 ± 12.10	18.79 ± 2.82
	MPR 0271	L	0.39 ± 0.06	0.09 ± 0.01	0.21 ± 0.03
		FP	13.04 ± 1.96	26.28 ± 3.94	5.91 ± 0.89
		SP	47.25 ± 7.09	115.57 ± 17.34	22.51 ± 3.38
	MPR 1501	L	3.03 ± 0.01	1.85 ± 0.03	1.26 ± 0.01
		FP	25.23 ± 3.78	37.38 ± 5.61	10.41 ± 1.56
		SP	55.34 ± 8.30	117.49 ± 17.62	28.24 ± 4.24
	MPR 1511	L	8.26 ± 1.24	2.21 ± 0.33	2.07 ± 0.31
		FP	13.09 ± 1.96	19.84 ± 2.98	6.49 ± 0.97
		SP	26.10 ± 3.91	60.43 ± 9.06	13.15 ± 1.97
	MPR 1531	L	0.11 ± 0.02	0.06 ± 0.01	0.06 ± 0.01
		FP	18.90 ± 2.84	22.86 ± 3.43	8.30 ± 1.24
		SP	17.13 ± 2.57	39.95 ± 5.99	10.54 ± 1.58
*Magnolia parent plants*	MOB 1511	L	0.70 ± 0.01	54.18 ± 2.76	5.55 ± 0.25
		FP	1.74 ± 0.26	271.63 ± 40.74	13.04 ± 1.96
		SP	4.70 ± 0.70	649.38 ± 97.41	30.99 ± 4.65
	MTR 1531	L	191.62 ± 28.74	4.03 ± 0.60	53.61 ± 8.04
		FP	252.26 ± 37.84	71.85 ± 10.78	83.16 ± 12.47
		SP	230.82 ± 34.62	269.31 ± 40.40	99.91 ± 14.99

^a^ MPR = *Magnolia × pruhoniciana*, MOB = *M. obovata*, MTR = *M. tripetala*, L = leaves, FP = flower petals, SP = sexual parts of plants, ± SD (standard deviation).

**Table 3:** Percentages of growth inhibition I (%) of *Staphylococcus aureus* by extracts from different parts of *Magnolia* plants in different concentrations ^a^.

	Plant	Part of Plant	I (%) for Concentration of Extract	
			125 μg·mL^−1^	62.5 μg·mL^−1^
*Magnolia hybrid plants*	MPR 0131	L	63.3 ± 3.7	54.3 ± 1.4
		FP	98.3 ± 3.2	84.9 ± 1.8
		SP	100.0 ± 7.2	81.7 ± 2.1
	MPR 0151	L	88.4 ± 2.2	81.4 ± 4.0
		FP	96.1 ± 1.8	13.9 ± 5.0
		SP	97.2 ± 3.3	77.1 ± 2.5
	MPR 0271	L	98.4 ± 1.8	62.5 ± 2.1
		FP	97.0 ± 0.8	15.0 ± 4.7
		SP	95.9 ± 0.5	62.5 ± 4.0
	MPR 1501	L	56.2 ± 1.2	57.1 ± 5.8
		FP	84.5 ± 2.1	83.9 ± 2.4
		SP	93.6 ± 4.6	87.9 ± 7.9
	MPR 1511	L	51.4 ± 3.0	53.5 ± 6.4
		FP	42.9 ± 4.4	0.0 ± 0.0
		SP	96.6 ± 1.4	82.7 ± 4.0
	MPR 1531	L	64.3 ± 3.6	34.5 ± 4.0
		FP	100.0 ± 2.0	37.2 ± 9.6
		SP	94.1 ± 0.3	48.8 ± 4.1
*Magnolia parent plants*	MOB 1511	L	80.4 ± 17.7	58.4 ± 11.3
		FP	65.1 ± 3.5	41.8 ± 6.7
		SP	98.4 ± 2.9	100.0 ± 4.0
	MTR 1531	L	66.6 ± 18.8	68.7 ± 3.8
		FP	65.4 ± 3.9	100.0 ± 4.6
		SP	92.0 ± 6.7	68.6 ± 35.6

^a^ MPR = *Magnolia* × *pruhoniciana*, MOB = *M. obovata*, MTR = *M. tripetala*, L = leaves, FP = flower petals, SP = sexual parts of plants, ± SD (standard deviation).

**Table 4:** MIC (μg·mL^−1^) and IC_50_ (μg·mL^−1^) of tested extracts for the bacterium *S. aureus* ^a^.

	Plant	Part of Plant	IC_50_ (μg·mL^−1^)	MIC ^b^ (μg·mL^−1^)
*Magnolia hybrid plants*	MPR 0131	L	-	>125
		FP	56.6 ± 0.8 ^c^	125 ± 6.3
		SP	46.9 ± 2.0 ^c^	125 ± 6.3
	MPR 0151	L	002D	125
		FP	-	>125
		SP	57.6 ± 0.1 ^c^	125 ± 6.3
	MPR 0271	L	62.9 ± 10.9 ^c^	125 ± 6.3
		FP	-	>125
		SP	-	>125
	MPR 1501	L	-	>125
		FP	-	>125
		SP	-	> 25
	MPR 1511	L	-	>125
		FP	-	>125
		SP	-	>125
	MPR 1531	L	-	>125
		FP	71.0 ± 5.4 ^c^	125 ± 6.3
		SP	-	>125
*Mgnolia parent plants*	MOB 1511	L	-	>125
		FP	-	>125
		SP	40.1 ± 7.4 ^c^	62.5 ± 3.1
	MTR 1531	L	-	>125
		FP	31.6 ± 0.2 ^c^	62.5 ± 3.1
		SP	60.2 ± 26.4 ^c^	62.5 ± 3.1

^a^ MPR = *Magnolia × pruhoniciana*, MOB = *M. obovata*, MTR = *M. tripetala*, L = leaves, FP = flower petals, SP = sexual parts of plants, ± SD (standard deviation), ^b^ MIC (vancomycin) = 1 μg·ml^−1^, ^c^ Significantly (*p* < 0.05) different from the control.

**Table 5:** Elution gradient.

Time (min)	Methanol	10 mM Formic Acid
2	5%	95%
45	90%	10%
50	100%	0%
60	100%	0%
65	5%	95%
